# Intracellular accommodation of bacteria, fungi, and oomycetes by plants analyzed using transmission electron microscopy

**DOI:** 10.1371/journal.ppat.1013780

**Published:** 2025-12-22

**Authors:** Isabella Gantner, Caroline Gutjahr, Martina K. Ried-Lasi, Andrea Cheradil, Julia Buchner, Martin Parniske, Andreas Klingl

**Affiliations:** 1 Faculty of Biology, LMU Munich, Martinsried, Germany; 2 Max Planck Institute of Molecular Plant Physiology, Potsdam, Germany; 3 Leibniz Institute of Plant Biochemistry, Halle, Germany; University of Tübingen: Eberhard Karls Universitat Tubingen, GERMANY

## Abstract

Transmission electron microscopy was the key for revealing structural similarities between intracellular plant-microbe interactions.

## Scientific background

The advent of high-resolution imaging using transmission electron microscopy (TEM) was instrumental to reveal previously undetectable details of sub-cellular interfaces between host cells and intracellular microorganisms [1,2]. While the resolution of light microscopy is restricted to 200 nm due to the diffraction limit of light, electron microscopy has the potential to reach a resolution of 1 nm or even below [3]. This imaging capability enabled detailed exploration of the cellular ultrastructure, and revealed subcellular features like membrane invaginations [4], the structure of organelles [5], and new organelle-like cell compartments [1]. Based on the comparative analysis of TEM images originating from different laboratories working on different plant-microbe interactions, it has been postulated more than 25 years ago, that all intracellular symbioses, featuring living microorganisms in living plant cells, share a series of common structural features [6]:

(1) Entire microorganisms, in case of bacteria or their hyphal extensions in case of fungi or oomycetes, are hosted inside a living plant cell [6]. (2) A plant-derived membrane separates the accommodated microorganism from the cytoplasm of the host plant cell [6]. Depending on the interaction, this membrane is called peribacteroid membrane (PBM) in the nitrogen-fixing root nodule symbiosis (RNS) [7], periarbuscular membrane (PAM) in arbuscular mycorrhiza (AM) [8], and perihaustorial or extrahaustorial membrane (PHM/EHM) around the haustorium formed by pathogenic fungi or oomycetes inside living plant cells [9]. (3) The presence of a space between perimicrobial membrane and the outermost layer of the microorganism (the outer membrane in case of rhizobial bacteria and the cell wall in case of oomycetes or fungi). Depending on the interaction, this space is called peribacterial space (PBS) [7], periarbuscular space (PAS) [10], and peri- or extrahaustorial space (PHS/EHS) [11].

In this Pearl, we present, explain, and compare the structural interfaces of three such intracellular interactions, documented by the same lab using the same equipment, microscope settings, and sample preparation methods, all carried out by the same scientist.

**Fig 1:**
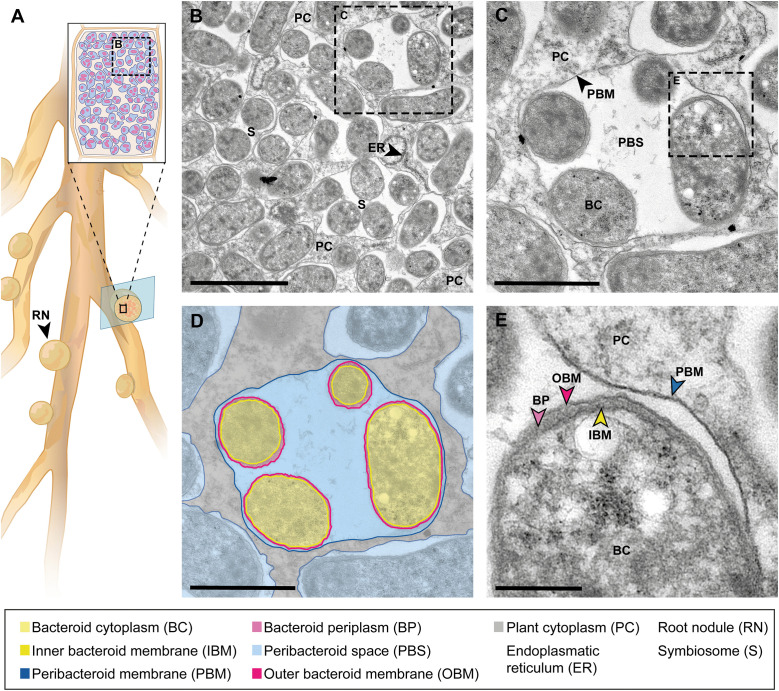
Ultrastructure of symbiosomes in the RNS between *Lotus japonicus* and *Mesorhizobium loti.* (**A)** Schematic drawing of a *L. japonicus* root with root nodules and a cross-section through a colonized nodule cell filled with symbiosomes. (**B)** TEM image of a densely colonized zone in a root nodule cell with several bacteroids packed in symbiosomes and surrounded by plant cytoplasm. In case of the bacterium *M. loti*, in symbiosis with *L. japonicus*, the bacteroid ultrastructure is similar to the free-living state [13,14]. The bacteroids maintain the rod shape with a length of around 1 µm. However, depending on whether the bacteroids were cut longitudinally or transversally, their visible shape can range from rod to round shaped. The slightly wavy appearance of the bacteroid membranes is caused by minimal shrinkage of the cells within chemical fixation. In some cases the bacteroid membranes are difficult to be recognized and to be separated from each other. Scale bar: 2.5 µm. **(C)** TEM image of a symbiosome and **(D)** corresponding schematic drawing. The symbiosome in C and D consists of four bacteroids, the PBS and the PBM. The symbiosome is surrounded by plant cell cytoplasm and other symbiosomes. The plant-derived PBM and the electron-translucent PBS represent the envelope of bacteroids. The bacteroid outer and inner membrane surrounds the cytoplasm of the *M. loti* cell and separate it from the surrounding PBS. Scale bar: 1 µm; **(E)** Close-up image of panel (C), with details of the plant-microbe interface and the membranes of both organisms. Scale bar: 250 nm.

**Fig 2:**
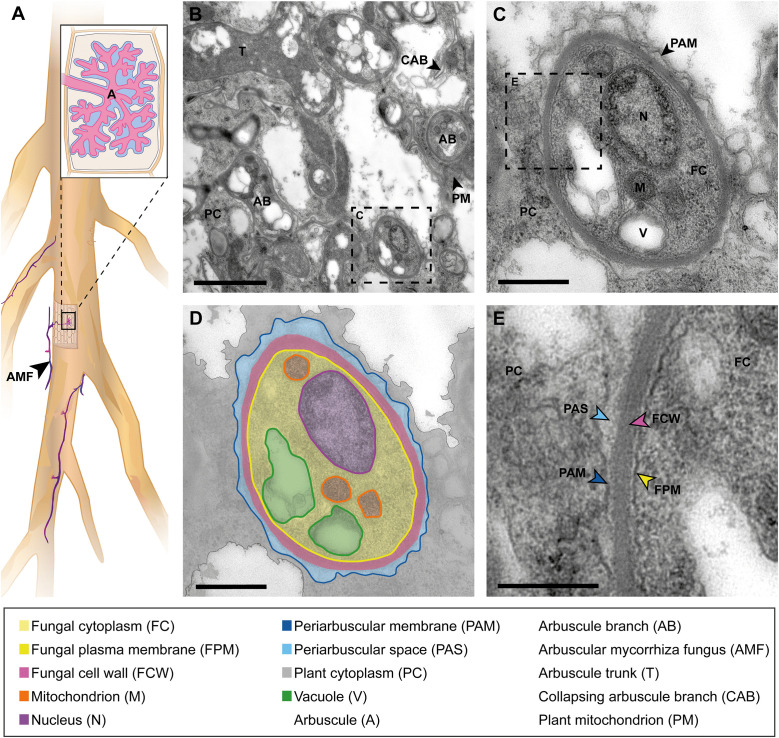
Ultrastructure of arbuscule branches formed by the AM fungus *Rhizophagus irregularis* in symbiosis with the legume host *Lotus japonicus.* **(A)** Schematic drawing of a *L. japonicus* root with hyphopodia an of AM fungus (AMF) and a hypha, penetrating the root surface. A longitudinal section through a root cortex cell inside the root tissue, hosting an arbuscule. **(B)** TEM image of a subcellular region of a sectioned host cell showing a part of an arbuscule, with a trunk and several branches in different sizes distributed in the plant cytoplasm and between plant cell organelles. In the TEM image, the arbuscule branches appear darker than the plant cell cytoplasm and can have different shapes depending on whether they were cut longitudinally or transversally. In this section, the trunk has been sectioned longitudinally, whereas most thick and fine branches appear as round shapes as they have been cut transversally. Collapsing arbuscule branches can be recognized as squeezed fungal structures with linear shapes*.* Scale bar: 2 µm; **(C)** TEM image of an arbuscular branch cross-section and **(D)** corresponding schematic drawing. The arbuscule is surrounded by a plant-derived PAM and PAS and a layer of plant cell cytoplasm. The slightly wavy appearance of the PAM is caused by minimal shrinkage of structures within chemical fixation. The arbuscule cell wall and the fungal plasma membrane envelopes the cytoplasm of *R. irregularis* and appears electron dense. The fungal cytoplasm contains a nucleus and mitochondria with electron-dense substructures and several bright vacuolar spaces. Scale bar: 0.5 µm. **(E)** Close-up of panel (C) with details of the plant-microbe interface layers (arrows) that are in close contact to each other. Scale bar: 250 nm.

**Fig 3:**
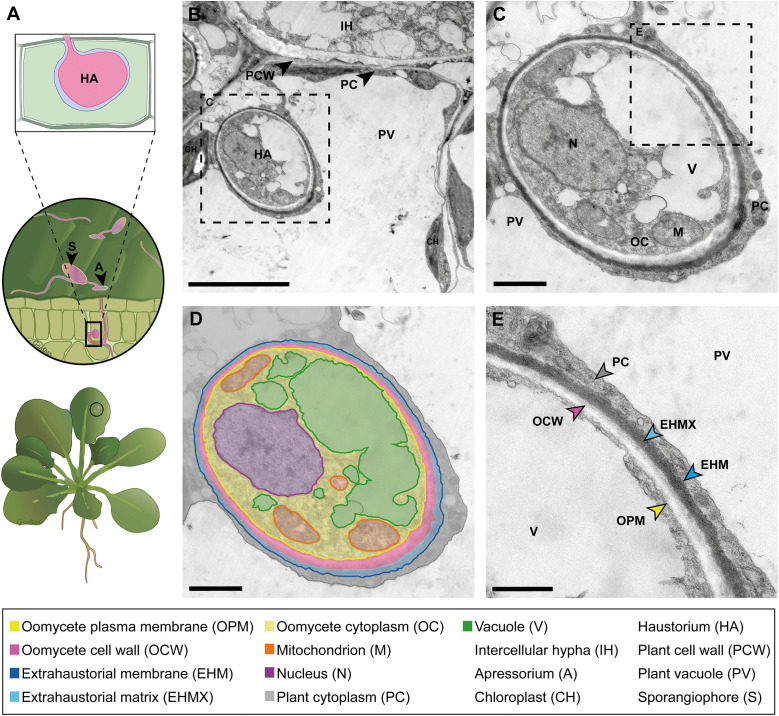
Ultrastructure of a haustorium of the downy mildew-causing oomycete *Hyaloperonospora arabidopsidis* in *Arabidopsis thaliana.* **(A)** Schematic drawing showing from bottom to top an *A. thaliana* plant, a magnification of a leaf surface colonized by *H. arabidopsidis,* and a longitudinal section of a palisade mesophyll leaf cell hosting a haustorium (HA). **(B)** TEM image of a cross-section of an oomycete intercellular hypha in the plant intercellular space and an oomycete haustorium inside the plant mesophyll cell surrounded by plant cytoplasm and a chloroplast. Scale bar: 5 µm; **(C)** TEM image of a haustorium in cross section and **(D)** corresponding schematic drawing. The haustorium is surrounded by a layer of plant cell cytoplasm, a plant-derived EHM, and the extrahaustorial matrix (EHMX). The EHMX, with a strong electron-dense appearance, is a carbohydrate-rich, gel-like layer, which lies between the haustorium and the EHM and mediates molecular exchange across this interface. The cytoplasm is surrounded by the plasma membrane and by the oomycete cell wall, a brighter, more electron-lucent appearing layer. The haustorial cytoplasm contains several bright vacuolar spaces, a nucleus, as well as mitochondria with small substructures of the cristae. The haustorium cell wall and the plasma membrane envelope the cytoplasm of the oomycete. Scale bar: 1 µm. **(E)** Close-up image of (C) with details of the plant-microbe interface layers (arrows), that are in direct contact to each other. Scale bar: 500 nm.

The preparation was carried out, following a protocol described previously [12]. All biological samples were chemically fixed in cacodylate buffer with 2.5% glutaraldehyde immediately after tissue harvesting, to crosslink proteins and stabilize cellular components. Samples were post-fixed with 1% osmium tetroxide for 1 h, which stabilizes and enhances electron density of lipids, and subsequently dehydrated using a graded acetone series from 10% to 100% combined with the addition of 1% uranyl acetate in the 20% acetone step. This heavy metal binds to phosphate groups, e.g., of nucleic acids and phospholipids, and improves their contrast by increasing electron density. The thick plant tissue was gradually infiltrated with Epon epoxy resin, ending with a 100% resin incubation step for 18 h. The resin-embedded samples were then polymerized at 63 °C for 24 h. After ultra-thin sectioning, images were acquired with a transmission electron microscope at an acceleration voltage of 80 kV. As examples for different plant-microbe interfaces in different plant species and tissues, we compared the nitrogen-fixing bacterium *Mesorhizobium loti* hosted inside a root nodule cell of the host plant *Lotus japonicus* (Fig 1), the AM fungus *Rhizophagus irregularis* forming an arbuscule inside a *L. japonicus* root cell (Fig 2), and the biotrophic oomycete *Hyaloperonospora arabidopsidis* forming a haustorium in a leaf cell of *Arabidopsis thaliana* (Fig 3).

## The ultrastructure of symbiosomes in the nitrogen-fixing root nodule symbiosis

Legumes benefit from a symbiosis (Fig 1) with nitrogen-fixing rhizobia, which can convert atmospheric nitrogen into ammonium and make it available to the host plant. In return, the host provides a carbon source, succinate, to the bacterial symbiont [14].

The colonization process of *L. japonicus* by *M. loti* is characterized by the formation of tubular structures called infection threads containing cell files of rhizobia. Infection threads form across the interior of host cells, and can connect opposite cell wall boundaries, enabling rhizobial passage from cell to cell, while root cortical cells divide to form a nodule primordium [15]. Inside the central tissue of the nodule, the bacteria are hosted within living plant cells inside organelle-like compartments, called symbiosomes [14], in which the bacteria are separated from the plant cytoplasm by a plant-derived PBM [6,14]. The term symbiosome was suggested by Roth, Jeon, and Stacey [16] to postulate a conceptually new organelle built from three main components, specifically the bacteroid (a term used to describe a rhizobial cell conceptually differentiated to exist as part of the symbiosome), the PBS, and the PBM in a non-lytic subcellular compartment [17]. By definition, a single symbiosome comprises the PBM surrounding the PBS and one longitudinally elongated (e.g., *Medicago*) or several (e.g., *Lotus*) bacteroids located in the PBS [18]. However, the bacteroid number per symbiosome and morphology is largely dictated by the host plant [13,14]. The bacteroids are surrounded by PBS, which contains a variety of compounds, including enzymes, proteins, and sugars, derived from plant and bacteroid origin [19,20]. However, the critical boundary between bacteroids and plant cytoplasm is the PBM, as it controls nutrient and signal exchange between the interacting partners [21].

## The ultrastructure of an arbuscule in arbuscular mycorrhiza symbiosis

AM is the name of a symbiosis formed between plants and fungi of the Glomeromycotina. AM fungal hyphae collect macro- and micro- nutrients, such as nitrogen and phosphate, from the soil and deliver them to the plant via arbuscules formed inside plant cells, thereby contributing to plant nutrition [22]. In return, plants provide the obligate biotrophic AM fungi with essential fatty acids that the fungus cannot synthesize on its own, and potentially sugars [23,24].

AM fungi enter the plant tissue in a plant-assisted intracellular colonization process (Fig 2A) [25]. In the cortex, in Arum-type symbioses, the fungal hyphae spread outside the host cells, in the apoplast along the longitudinal axis of the root, and penetrate into individual host cells to form a highly branched tree-shaped structure called arbuscule (lat. arbuscula = small tree) [26]. The arbuscule comprises a thick trunk, first-order thick branches, and higher-order fine branches. An individual arbuscules lives for only about 2–3 days at maturity, then it collapses gradually and disappears from the cell [27,28].

Arbuscules are surrounded by a plant-derived peri-arbuscular membrane (PAM). The PAM can be divided into two domains, one surrounding the trunk and thick branches and the other surrounding the fine branches [29]. Most of the nutrient exchange between AMF and plant hosts occurs between arbuscules and their accommodating root cortex cells, i.e., across the PAM [30]. Nutrients also need to cross the PAS, an apoplastic space between the PAM and the fungal cell wall.

## The ultrastructure of an oomycete haustorium

The obligate biotrophic oomycete *Hyaloperonospora arabidopsidis* (Hpa) is the causal agent of downy-mildew disease in *A. thaliana* (Fig 3). Oomycetes are filamentous eukaryotes with fungus-like growth habits, notorious for causing devastating crop losses [31].

During infection, the oomycete produces germ tubes, that penetrate the plant’s outer surface via appressorium-like structures or through natural openings such as stomata and spread through the plant tissue by intercellular hyphae [32]. Once they breach the host cell wall, hyphae differentiate into haustoria, specialized structures that reside inside the host cell but remain separated by an enveloping EHM from the host cytoplasm. The EHM is a unique, plant-derived compartment with distinct protein and lipid composition [9]. The extrahaustorial matrix (EHMX) lies between the haustorium and the EHM. The EHM is thought to mediate molecular exchange between the two organisms [33]. However, in contrast to RNS and AM, direct evidence for nutrient exchange in the *Hpa–Arabidopsis* interaction remains limited. Haustoria are potentially involved in osmotrophic feeding [34] and effector secretion to manipulate host immunity and promote infection [35–37]. Thus, the haustorial interface is a dynamic battleground for molecular exchange and is fundamentally shaping plant–pathogen interactions.

## Conclusion

We performed ultrastructural analysis of three different intracellular plant–microbe interactions covering three different kingdoms of microorganisms hosted by two different angiosperm host plants and grown in three different laboratories, but by performing the sample preparation and the electron microscopy in the same laboratory by the same person. We presented annotated images highlighting the postulate made 25 years ago, that structural similarities exist between symbiosomes, arbuscules, and haustoria [6]. We present the common and unifying feature of these interactions, the presence of a plant-derived membrane that envelops the microbe and separates it from the plant cytoplasm. We illustrated that these similarities exist regardless of the plant host species, tissue type, or whether the microbe is prokaryotic or eukaryotic, and its lifestyle, symbiotic or pathogenic. However, there are also structural differences between the presented plant–microbe interactions. In comparison to prokaryotic rhizobacteria in RNS, the eukaryotic AM fungi and oomycetes possess organelles in their cytoplasm. Furthermore, AM fungi and oomycetes exhibit a thick cell wall, while rhizobia are, in contrast to this, enclosed by the peptidoglycan layer in the periplasm and an outer membrane, that conceptually increases the distance between the plant host and the accommodated microbe. Both must be overcome by diffusion during transport and communication between the microbe and its host [38]. The images presented in this comparative analysis also visualize profound differences for the interface spaces, PBS, PAS, and EHMX. The PBS appears rather broad and inhomogeneous in RNS, while the PAS in AM is relatively thinner and denser. The EHMX in the interaction between oomycete and *A. thaliana* appears extremely reduced and with electron-dense deposits, a feature also described for the extrahaustorial matrix of pathogenic fungi [39]. These differences in structure, optical density, and contrast agent accumulation hint towards a significant difference in the composition or concentration of substances in the PBS, PAS, and EHMX. This could be due to the different plant host and microbe species presented in this comparison, or is related to the symbiotic and pathogenic lifestyles of the organisms.

In addition, we confirmed that all these interactions are indeed featuring living microorganisms hosted by living plant cells. This conclusion is based on structural features such as the presence of organelles inside the microorganism and the plant cell: rough endoplasmatic reticulum in Fig 1B, plant mitochondria in Fig 2B and chloroplasts in Fig 3B. The capabilities of electron microscopy to capture such detailed observations also opens possibilities for further comparative investigations, including ultrastructural analyses of organelles in infected versus uninfected host cells, as well as studies of microbial morphology both inter- and intracellular in the host tissue. Moreover, advancing such comparative studies by integrating electron microscopy with complementary imaging techniques and molecular biology approaches can bridge knowledge gaps and yield deeper insights into the similarities and differences underlying these interactions.
